# Respectful maternity care in Israel during the Covid-19 pandemic: a cross-sectional study of associations between childbirth care practices and women’s perceptions of care

**DOI:** 10.1186/s12884-023-06030-5

**Published:** 2024-01-10

**Authors:** Hagar Palgi-Hacker, Emma Sacks, Megan Landry

**Affiliations:** 1 Milken Institute School of Public Health, The George Washington University, 950 New Hampshire Avenue NW, Washington, D.C, 20052 USA; 2 Department of International Health, Johns Hopkins School of Public Health, 615 N. Wolfe St, E8011, Baltimore, MD 21205 USA

**Keywords:** Maternal health, Childbirth, Quality of care, Mistreatment, Survey, Disrespect and abuse, Israel, Covid-19

## Abstract

**Background:**

Respectful maternity care is a crucial part of quality care and is associated with better health outcomes. Early in the Covid-19 pandemic, reports from across the world indicated that infection containment measures were often implemented in ways that resulted in disrespectful care of women during facility-based childbirths in violation of evidence-based practices. This study aimed to explore the associations between childbirth care practices and perceptions of care as satisfactory and respectful among women who delivered in Israeli hospitals during the first six months of the Covid-19 pandemic.

**Methods:**

A cross-sectional self-administered online survey was conducted to explore women’s perceptions of maternity care using an adapted version of the WHO Community Survey Tool for measuring how women are treated during facility-based childbirth. Multivariate logistic regression models evaluated the associations between sociodemographic characteristics, obstetric information, and measurements of childbirth experiences and women’s perceptions of receiving respectful and satisfactory care.

**Results:**

The responses of 981 women were included in the analysis. While the majority of women perceived the care they received as both respectful (86.54%) and satisfactory (80.22%), almost 3 in 4 women (72.68%) reported experiencing at least one type of disrespectful care. Positive communication with the medical staff and respect for autonomy were associated with a more positive birth experience for women. Women were more likely to perceive their care as respectful if they did not feel ignored (AOR = 40.11;22.87–70.34). Perception of satisfactory care was more likely among women who had the opportunity to discuss preferences with the medical staff (AOR = 10.15; 6.93–14.86). Having Covid-19 procedures explained increased the likelihood of reporting respectful and satisfactory care (AOR = 2.89;1.91–4.36; AOR = 2.83;2.01–4).

**Conclusion:**

Understanding which care practices are associated with women’s perceptions of care at facility-based births is critical to ensuring quality care. The findings of this study can inform future work and research aimed at enhancing respectful maternity care during times of crisis and beyond.

## Plain English Summary

The Covid-19 pandemic affected health services around the world, including maternity care. At the beginning of the pandemic, many women reported being mistreated or receiving disrespectful care during childbirth in health facilities. This study investigated how the way women were taken care of while giving birth in Israeli hospitals during the first six months of the Covid-19 pandemic was connected to their satisfaction and perception of being treated well. The study used a survey developed by the World Health Organization. The study found that most of the 981 women who answered the survey perceived the care they received as both satisfactory and respectful. However, almost 3 of 4 of the women in the study said they experienced at least one kind of care practice that was not respectful. The study also found that women were more likely to think their care was satisfactory or respectful if they had positive communications with the medical staff, and if they did not feel the medical staff ignored them. This study can be used to understand what helps women perceive their care as satisfactory and respectful and promote more positive birth experiences in Israel.

## Background

Increased global attention has been given in recent years to women’s experiences of care during childbirth [1]. Shifting from focusing solely on childbirth-related morbidity and mortality, researchers began systematically documenting the mistreatment of women during facility-based births worldwide [2]. Mistreatment of women and other birthing people during childbirth can create a negative childbirth experience [3], is associated with immediate and lasting effects on the mental health of the woman [4], and can harm the initiation or continuation of breastfeeding, [5] and the mother-baby bonding in the postpartum period [6].

In support of advancing research on childbirth experiences, World Health Organization (WHO) researchers developed evidence-based typology and operational definitions of mistreatment of women during facility-based childbirth across seven themes: 1) physical abuse; 2) sexual abuse; 3) verbal abuse; 4) stigma and discrimination; 5) failure to meet professional standards of care; 6) poor rapport between women and providers; and 7) health system conditions and constraints [7]. This typology served the WHO researchers in developing and validating labor observation and community survey tools to measure how women are treated during facility-based births in various locations [8]. Mistreatment during childbirth constitutes a significant component of disrespectful care, which also encompasses instances where there is a failure to provide the expected level of respectful care. While eliminating mistreatment is crucial to promoting respectful care, respectful care extends beyond it [9]. Respectful maternity care is defined by WHO as “Care organized for and provided to all women in a manner that maintains their dignity, privacy and confidentiality, ensures freedom from harm and mistreatment, and enables informed choice and continuous support during labour and childbirth.“ (WHO recommendations: intrapartum care for a positive childbirth experience [10]). Respectful maternity care is an essential element of WHO’s woman-centered care recommendations for a positive childbirth experience and standards for improving quality of maternal care [11].

The implementation of respectful maternity care in birth facilities largely depends on the context of the health system. The outbreak of the Covid-19 pandemic brought sudden and dramatic changes to health systems globally [12]. As the novel coronavirus (SARS-CoV-2) rapidly spread around the world, governments responded with various containment mechanisms. Health systems carried the double burden of functioning under these measures and caring for large numbers of people infected with the virus, all with limited data [13]. The response mechanisms and uncertainties in the first few months of the pandemic led to significant changes in the provision of maternity care [14–17].

Reports quickly began to emerge suggesting that Covid-19-related policies and practices were leading to disrespectful care of women during childbirth, including bans on evidence-based practices, such as the presence of birth companions and non-separation of newborns from their mothers, as well as coerced medical procedures [18, 19]. As the WHO began providing guidelines for the provision of maternity care during the pandemic in March 2020 [20], advocates encouraged health facilities to adopt policies that both support respectful care and protect the safety of all stakeholders [21–23].

Israel saw a rapid spread of Covid-19 early on and has had one of the highest per capita case rates in the world [24], requiring hospitals to quickly adapt to the changing circumstances. The Ministry of Health, which oversees the Israeli national healthcare system, provided guidelines instructing hospitals on how to best deliver maternity care during Covid-19 [25]. In these guidelines, hospitals were instructed to keep mothers and newborns together as before, unless the mother tested positive for Covid-19 while the newborn tested negative. According to the guidelines, even in this latter case, a mother could choose to have her newborn stay with her and continue breastfeeding or provide pumped milk, with necessary precautions such as mask-wearing. In addition, hospitals were instructed to continue to allow fathers and other companions to enter hospitals, including nurseries and neonatal intensive care units, as long as they were not suspected Covid-19 cases and were wearing a mask. Despite these guidelines, women reported harmful practices were occuring [26]. Media reports suggested that birthing women, regardless of their Covid-19 infection status, encountered various challenges and limitations during childbirth, including limiting birth companionship and rooming-in, and restrictions on breastfeeding [27]. Women who tested positive for Covid-19, as well as some whose Covid status was unknown, also told of lack of privacy, forced separation from their newborns for days, and neglectful care that included limited access to medical care, supplies, and emotional support during childbirth and in the immediate postpartum period [28].

Israel has the highest fertility rate among the Organization for Economic Co-operation and Development (OECD) countries [29]. At the same time, Israel’s infant mortality rate is three per 1,000 live births [30], and its maternal mortality ratio is three per 100,000 live births [31]. National health insurance covers extensive antenatal care, as well as all medical costs associated with childbirth in hospitals [32], where 99% of births take place [33]. Midwives attend to the majority of births but are supervised by obstetricians, who also attend to medically complicated births and perform cesarean sections (C-sections) [34]. The Ministry of Health has stated it is not supportive of home births and non-hospital birth centres and has regulated strict guidelines for medical professionals attending such births [35]. Grassroots efforts to expand the option and expense coverage for home births have been ongoing [36].

Limited systematic research exists on respectful maternity care in Israel before the Covid-19 pandemic, and the data available is limited to reporting disrespectful care practices. Reports from advocates, the media, and social media, reveal that multiple types of disrespectful practices occur in hospital maternity wards. In a 2019 report submitted to the United Nations Special Rapporteur on violence against women, the Israeli grassroots organization Women Call for Birth (Now called the Birth Freedom Israel Movement) reviewed the available data on disrespectful care during childbirth in Israel [37]. The report summarized the complaints received on the organization’s hotline, noting that over a period of two years, 670 complaints of disrespectful care during childbirth were reported. The complaints focused mainly on poor rapport with providers, lack of consent for procedures, and verbal or physical abuse. The report also included preliminary data from an unpublished 2018 survey on maternity care experiences in Israel. The survey's initial findings indicate that approximately 30% of the 3,204 respondents reported that informed consent prior to procedures was only obtained from them from a “moderate… to little” extent, or not at all. A similar percentage of respondents shared that their choices were only respected to a moderate to no extent. Recently, a study exploring women’s perceptions of gynecologic examinations in Israel found that harmful practices were occurring. Among the study’s 6,508 participants, 75.9% reported they did not receive a satisfactory explanation prior to being examined. In addition, a lack of privacy was identified by more than half of participants, with 65.4% reporting not being offered a cover during examinations, and 33% indicating no curtain was present in the examination room [38].

## Methods

### Study aims

This study aimed to explore the associations between childbirth care practices and perceptions of care as satisfactory and respectful among women who delivered in Israeli hospitals during the first six months of Covid-19 pandemic (March 1, 2020, to August 31, 2020). We hypothesized that women who experienced disrespectful care or Covid-19 related restrictions were less likely to perceive the care they received as satisfactory and respectful.

### Study design

This is a cross-sectional quantitative study that utilized a self-administered survey. Participants in the survey were women who delivered in any Israeli hospital during the first six months of the Covid-19 pandemic (March 1, 2020 to August 31, 2020).

#### Survey tool

The study survey tool was adapted from the WHO’s Community Survey Tool (CST) [8]. The CST was chosen because it covered multiple respectful and disrespectful childbirth care practices, allowing a comprehensive exploration of the association between these practices and perceptions of care.

The survey was adapted to the context of this study in several ways. First, the WHO survey was developed to be administered by a community research team. To enable the survey to be self-administered online due to pandemic constraints, only the core care experiences questions were included in the adapted version. These questions were then condensed (e.g., instead of asking separately about types of physical violence, a single question was formed with the option of selecting multiple answers). Second, questions about experiences deemed unlikely in the Israeli context as compared to the lower-income settings where the CST was first tested, were removed (e.g., bribes to facility staff, bed sharing with other patients, and availability of water or electricity). Third, Covid-19-specific questions were added to the survey. Finally, the demographic questions were adapted to align with the questions of the Israeli census. The adapted survey consisted of 54 questions (excluding screening questions) across four parts: obstetric characteristics, birth experiences, perceptions of care, and sociodemographic information. In the next step, the survey was translated from its original language of English to Hebrew, and then back translated to Hebrew by an additional translator to verify the accuracy of the translation. Cognitive interviews were held with three women who met the eligibility criteria to assess their comprehension of the survey questions, response options, and overall survey flow. The survey was then sent to ten additional women as a pilot.

### Settings, participants, and study size

#### Eligibility criteria

Women were eligible to participate in the study if they were 18 and older, gave birth in an Israeli hospital between March 1, 2020 and August 31, 2020, and provided written consent for participation after reading and approving an informed consent form.

#### Sample size

The minimum sample size was calculated to be 383 women, using an estimated number of births in Israel during a 6-month period of 90,000 [29]. The sample size was based on a confidence interval of 95%, a 5% margin of error, and a standard deviation of 0.5.

#### Recruitment and sampling

Convenience and snowball sampling were used in this study through social media platforms, primarily Facebook. A link to the survey was shared in groups and ads and could be forwarded. More than 30 groups were contacted and agreed to share the recruitment post. These groups included Israeli women and mothers' groups, as well as pregnant mother groups, and groups dedicated to delivery mode (C-section, natural birth, active labor) and breastfeeding, and comprised sub-populations of various categories, such as those of particular geographic location, ethnicity, and religion (Ethiopian, Arab, Russian, orthodox, and ultra-orthodox Jewish). Other Covid-19-related studies have been successful in using similar recruitment and sampling strategies [39, 40]. A recruitment post in Hebrew and Arabic was posted on a designated Facebook page, and shared on various Facebook groups. Hard-to-reach populations, including Arab, Palestinian, and Bedouin women, Ethiopian women, and Jewish Ultra-Orthodox women, were recruited using paid ads on Facebook and Instagram. After completing the survey, participants who wished to participate in a raffle to win one of four baby gift baskets, valued at approximately $50 USD each, were prompted to click on a new link. The new link led to a new survey page where participants were asked to provide their contact information. To maintain the anonymity of participants, the raffle survey was not associated with the study survey.

#### Data collection

The survey was an online, self-administered questionnaire in Hebrew, and was hosted on SurveyMonkey. The survey was open for responses between January 7, 2021, and January 18, 2021. No personally identifying information was collected, and participants remained anonymous.

In this study, 1287 women entered the survey and were screened for eligibility. A total of 119 women were excluded for ineligibility, and an additional 106 women did not complete the screening questions. An additional 81 women did not complete the survey. The responses of 981 women were included in the analysis.

### Data analysis

#### Outcome variables

The two outcome variables in this study were: 1) overall satisfaction of care received (“Overall, I am satisfied with the care I received during my stay at the hospital for childbirth”); and 2) feeling of being treated with respect (“I feel that the doctors, nurses, and midwives treated me with respect”). Participants answered both questions using a Likert scale coded into binary variables (yes = strongly agree/agree, and no = strongly disagree/disagree/neutral), per the WHO analysis of the community survey tool [41]. Multi-category predictor variables were also aggregated into binary variables to facilitate statistical analysis. The answers “prefer not to answer”, “not sure”, and “not relevant” were coded as missing and excluded from further analysis.

#### Statistical analysis

Survey data were cleaned prior to data analysis. Codes were developed based on the definitions provided in the WHO typology [7]. Open-ended comments were categorized based on the codes; comments that mentioned multiple experiences were coded in each relevant category.

Descriptive statistics and Pearson’s Chi-square test were used for bivariate analysis to describe sociodemographic and obstetric characteristics and childbirth care practices, and to present the frequency and percentages of satisfactory and respectful care by these variables. Pearson’s Chi-square test was used to explore the relationship between the predictor and the outcome variables. Variables that were found to have a statistically significant association with at least one of the outcome variables (*p*-value ≤ 0.05) were then entered into a multivariable model.

Based on the results of the bivariate analysis, separate multivariable logistic regression models were constructed to evaluate the association between each childbirth care practice, measurements of perception of care (using a Likert-like scale), and Covid-19-related characteristics and experiences, and the outcome variables. All models were adjusted for age, country of birth, marital status, education, income, parity, religion, and level of religiosity. Models were also adjusted for the level of pregnancy risk (high or low), mode of delivery (vaginal or C-section), and the occurrence of any intervention. Model fitness was examined using the Hosmer–Lemeshow test, and multicollinearity was checked using the variance inflation factor test. Predictor variables associated with the outcome variables were identified using an adjusted odds ratio (AOR) with 95% confidence intervals (CI), and statistical significance of p-value ≤ 0.05. Data analysis was conducted using Stata V14.2. We adhere to STROBE (Strengthening the Reporting of Observational Studies in Epidemiology) guidelines for reporting of observational studies.

### Ethical approval

The George Washington University Committee on Human Research, Institutional Review Board (IRB) approved the study (IRB# NCR203064). Informed consent was obtained from each participant at the beginning of the survey. No personally identifiable information was collected and the participants remained anonymous.

## Results

### Sociodemographic characteristics

A total of 981 responses from women who completed the survey are included in this analysis. Table 1 presents the sociodemographic characteristics of the study participants. The mean age of participants was 31.3 (SD ± 0.14), with the vast majority (81.65%) between the ages of 27 and 38. The mean age of participants giving birth for the first time was 29.81 (SD ± 4.01). Almost all study participants were married or in a common-law marriage at the time of childbirth (94.8%). Half of participants were primiparous (50.46%). The majority of participants were Jewish (83.59%), followed by Muslim (11.01%) and Christian (3.57%), and about half of participants were secular (52.8%). The majority of participants held a bachelor’s degree (51.38%), and half of participants reported their family income at below or significantly below the average national income per household (33.67% and 16.58%, respectively). Only limited associations between sociodemographic characteristics and perceptions of care were found. Most notably, women who were primiparous reported significantly lower rates of perceiving their care as respectful and satisfactory compared to women who had two or more children (16.36% compared to 10.49%, and 25.05% compared to 14.4%, respectively).﻿

**Table 1:** Sociodemographic characteristics by perception of respectful and satisfactory care

	**Sociodemographic** **Characteristics**		**Respectful Care**		**Satisfactory Care**	
	*N* = 981	%	Yes N (%)	No N (%)	Yes N (%)	No N (%)
**Total**			849 (86.54)	132 (13.46)	787 (80.22)	194 (19.78)
**Age**						
20–24 years old	54	5.5	45 (83.33)	9 (16.67)	44 (81.48)	10 (18.52)
25–29 years old	294	29.97	251 (85.37)	43 (14.63)	222 (75.51)	72 (24.49)
30–34 years old	416	42.41	371 (89.18)	45 (10.82)	343 (82.45)	73 (17.55)
35–39 years old	172	17.53	144 (83.72)	28 (16.28)	141 (81.98)	31 (18.02)
40 or above years old	45	4.59	38 (84.44)	7 (15.56)	37 (82.22)	8 (17.78)
**Country of Birth**						
Israel	846	86.24	736 (87)	110 (13)	682 (80.61)	164 (19.39)
Former USSR	78	7.95	66 (84.62)	12 (15.38)	58 (74.36)	20 (25.64)
Ethiopia	23	2.34	21 (91.3)	2 (8.7)	20 (86.96)	3 (13.04)
Other	34	3.47	26 (76.47)	8 (23.53)	27 (79.41)	7 (20.59)
**Personal Status**						
Married	868	88.48	755 (86.98)*	113 (13.02)*	696 (80.18)	172 (19.82)
In Common-Law Marriage	62	6.32	48 (77.42)*	14 (22.58)*	52 (83.87)	10 (16.13)
In a Relationship	21	2.14	21 (100)*	0 (0)*	15 (71.43)	6 (28.57)
Single	18	1.83	16 (88.89)*	2 (11.11)*	15 (83.33)	3 (16.67)
Divorced	7	0.71	4 (57.14)*	3 (42.86)*	5 (71.43)	2 (28.57)
Other	5	0.5	5 (100)*	0 (0)*	4 (80)	1 (20)
Parity*						
First Pregnancy	495	50.46	414 (83.64)	81 (16.36)	371 (74.95)	124 (25.05)
Two or More Previous Pregnancies	486	49.54	435 (89.51)	51 (10.49)	416 (85.60)	70 (14.4)
**Religion**						
Jewish	820	83.59	705 (85.98)	115 (14.02)	654 (79.76)	166 (20.24)
Muslim	108	11.01	96 (88.89)	12 (11.11)	90 (83.33)	18 (16.67)
Christian	35	3.57	31 (88.57)	4 (11.43)	29 (82.86)	6 (17.14)
Other	18	1.83	17 (94.44)	1 (5.56)	14 (77.78)	4 (22.22)
**Level of Religiosity**	*N* = 960					
Secular	518	53.96	437 (84.36)	81 (15.64)	410 (79.15)	108 (20.85)
Traditional	258	26.88	229 (88.76)	29 (11.24)	210 (81.4)	48 (18.6)
Orthodox	159	16.56	145 (91.19)	14 (8.81)	127 (79.87)	32 (20.13)
Ultra Orthodox	25	2.6	22 (88)	3 (12)	21 (84)	4 (16)
**Education**	*N* = 974					
Less than 12 Schooling Years	7	0.72	6 (85.71)	1 (14.29)	6 (85.71)	1 (14.29)
12 Schooling Years, High School Diploma, or Professional Certificate	211	21.66	181 (85.78)	30 (14.22)	173 (81.99)	38 (18.01)
Bachelor's Degree	504	51.75	435 (86.31)	69 (13.69)	396 (78.57)	108 (21.43)
Master's or PhD degree	252	25.87	220 (87.3)	32 (12.7)	207 (82.14)	45 (17.86)
**Income**	*N* = 932					
Significantly Below Average	162	17.38	143 (88.27)	19 (11.73)	135 (83.33)	27 (16.67)
Below Average	329	35.3	288 (87.54)	41 (12.46)	252 (76.6)	77 (23.4)
Average Income	251	26.93	224 (89.24)	27 (10.76)	206 (82.07)	45 (17.93)
Above Average	168	18.03	137 (81.55)	31 (18.45)	137 (81.55)	31 (18.45)
Significantly Above Average	22	2.36	17 (77.27)	5 (22.73)	16 (72.73)	6 (27.27)

^*^Statistically significant at *P* ≤ 0.05 based on chi-squared tests

### Obstetric characteristics

The majority of participants delivered at or after 37 weeks of pregnancy term (96.64%). Vaginal births accounted for 79.20% of deliveries, with only midwives present at most vaginal births (69.76%). The remaining vaginal births (30.24%) were attended by an obstetrician alone or with a midwife. A fifth of participants underwent a C-Sect. (20.8%), and more than half were emergency surgeries (56.37%). More than half of participants (62.49%) experienced at least one of the following interventions: labor induction (34.05%), labor augmentation (43.02%), episiotomy (20.9%), or use of forceps or vacuum (8.46%). Table 2 presents the obstetric characteristics of the study participants. While having a doctor participate in vaginal births resulted in a higher proportion of dissatisfaction among participants, this finding was non-significant once adjusted for instrumental birth.

**Table 2:** Obstetric characteristics by perception of respectful and satisfactory care

	**Obstetric Characteristics**		**Respectful Care**		**Satisfactory Care**	
	*N* = 981	%	Yes N(%)	No N(%)	Yes N(%)	No N(%)
**Pregnancy Risk Level**						
High Risk Pregnancy	237	24.16	205 (86.5)	32 (13.5)	191 (80.59)	46 (19.41)
Low Risk Pregnancy	744	75.84	644 (86.56)	100 (13.44)	596 (80.11)	148 (19.89)
**Mode of Delivery**						
** Vaginal Birth**	777	79.2	676 (87)	101 (13)	629 (80.95)	148 (19.05)
Midwife only	542	69.76	475 (87.64)	67 (12.36)	450 (83.03)*	92 (16.97)*
Doctor participated in the delivery	235	30.24	201 (85.53)	34 (14.47)	179 (76.17)*	56 (23.38)*
** C-Section**	204	20.8	173 (84.8)	31 (15.2)	158 (77.45)	46 (22.55)
Emergency C-Section	115	56.37	96 (83.48)	19 (16.52)	82 (71.3)*	33 (28.7)*
Elective C-Section	89	43.63	77 (86.52)	12 (13.48)	76 (85.39)*	13 (14.61)*
**Interventions**						
At least one intervention	613	62.49	522 (85.15)	91 (14.85)	476 (77.65)*	137 (22.35)*
No intervention	368	37.51	327 (88.86)	41 (11.14)	311 (84.51)*	57 (15.49)*
Episiotomy	205	79.1	174 (84.88)	31 (15.12)	151 (73.66)*	54 (26.34)*
No Episiotomy	776	20.9	675 (86.98)	101 (13.02)	636 (81.96)*	140 (18.04)*
Forceps or Vacuum	83	8.46	71 (85.54)	12 (14.46)	58 (69.88)*	25 (30.12)*
No Forceps or Vacuum	898	91.54	778 (86.64)	120 (13.36)	729 (81.18)*	169 (18.82)*
Induction	334	34.05	290 (86.83)	44 (13.17)	263 (78.74)	71 (21.26)
No Induction	647	65.95	559 (86.4)	88 (13.6)	524 (80.99)	123 (19.01)
Augmentation	422	43.02	359 (85.07)	63 (14.93)	318 (75.36)*	104 (24.64)*
No Augmentation	559	56.98	490 (87.66)	69 (12.34)	469 (83.9)*	90 (16.1)*
*Statistically significant at *P* ≤ 0.05						

### Childbirth care practices 

More than two-thirds of the study participants (72.68%) reported experiencing at least one disrespectful care practice. The most frequent types of disrespectful care practices reported by participants were not being allowed to birth in their preferred position (41.57%), not making joint decisions with the medical staff regarding care (34.45%), and not having the opportunity to discuss preferences or requests with the medical staff (31.6%). Nearly a fifth of participants reported the medical staff did not always ask for their consent before procedures (19.78%). Of the participants, 18.04% felt that their privacy was not always respected during their time at the hospital, and 12.03% felt ignored by the medical staff. Physical force by the medical staff (fundal pressure, being held down on the bed, or during vaginal exam) was reported by 6.12% of participants, with fewer reporting verbal violence or negative comments (4.38% and 1.33%, respectively). Table 3 includes the frequencies and percentages of childbirth care practices and associations with perception of care variables. ﻿Participants who did not experience any use of physical force were three times more likely to perceive the care they received as respectful (adjusted odds ratio (AOR) 2.61, 95% CI 1.5 to 5.25), and more than three times more likely to perceive it as satisfactory (AOR 3.55, 95% CI 1.93 to 6.5). Lack of verbal violence was associated with a greater likelihood of reporting both respectful care (AOR 7.69, 95% CI 3.7 to 15.99) and satisfactory care (AOR 4.4, 95% CI 2.16 to 8.93). The absence of negative comments from staff was associated with an increased likelihood of reporting respectful care (AOR 4.91, 95% CI 1.25 to 19.23), and satisfactory care (AOR 2.21, 95% CI 0.59 to 8.3). Being allowed to birth in the preferred position doubled women’s perception of respectful and satisfactory care (AOR 2.14, 95% CI 1.32 to 3.47 and 2.07, 95% CI 1.39 to 3.08, respectively). Care practices related to the newborn were also associated with perceptions of respectful and satisfactory care. Participants who were not separated from their newborns were almost twice as likely to report respectful care (AOR 1.76, 95% CI 1.15 to 2.71) and be satisfied with their care (AOR 2.34, 95% CI 1.63 to 3.37). Similarly, being able to breastfeed on demand increased the likelihood of perceiving care as respectful (AOR 1.83, 95% CI 1.15 to 2.91) and as satisfactory (AOR 2.14, 95% CI 1.44 to 3.16). Associations between respectful care practices and perceptions of the care as respectful or satisfactory were also found. Receiving an explanation and consenting prior to examinations and procedures were both positively associated with perceptions of respectful (AOR 4.94, 95% CI 3.16 to 7.7 and 3.94, 95% CI 2.58 to 6.02, respectively) and satisfactory care (AOR 5.28, 95% CI 3.54 to 7.87 and AOR 3.55, 95% CI 2.44 to 5.16, respectively). Having privacy respected increased the likelihood of participants’ report of respectful and satisfactory care (AOR 7.73, 95% CI 5 to 11.95 and AOR 5.75, 95% CI 3.91 to 8.48, respectively). Measures related to decision-making were also positively associated with perceptions of respect and satisfaction. Participants were 10 times more likely to perceive their care as respectful (AOR 9.52, 95% CI 5.99 to 15.12) and satisfactory (AOR 10.15, 95% CI 6.93 to 14.86) if they had an opportunity to discuss their preferences and requests with the medical staff. The perception of joint decision-making with the medical staff increased the likelihood of reporting respectful care by six times (AOR 6.11, 95% CI 3.93 to 9.5) and satisfactory care by nearly eight times (AOR 7.66, 95% CI 5.27 to 11.15). Reporting not feeling ignored by the medical staff increased the likelihood of perceiving care as respectful and satisfactory more than 40 times compared to reporting feeling ignored (AOR 40.11, 95% CI 22.87 to 70.34, and AOR 43.2, 95% CI 24.21 to 77.22, respectively). No statistically significant association was found between being allowed a birth companion of choice and perceptions of respectful or satisfactory care (AOR 0.86, 95% CI 0.39 to 1.88 and AOR 1.23, 95% CI 0.65 to 2.33, respectively).

**Table 3:** Childbirth experiences and association with perceptions of care as respectful and satisfactory

	**Total**		**Respectful Care**			**Satisfactory Care**		
	*N*	%	Yes N(%)	No N(%)	AOR (95% CI)	Yes N(%)	No N(%)	AOR (95% CI)
Any Physical Force*								
Yes	60	6.12	43 (71.67)	17 (28.33)	1	32 (53.33)	28 (46.67)	1
No	921	93.88	806 (87.51)	115 (12.49)	**2.61 (1.3 to 5.25)**	755 (81.98)	166 (18.02)	**3.55 (1.93 to 6.5)**
Any Verbal Violence *								
Yes	43	4.38	19 (44.19)	24 (55.81)	1	20 (46.51)	23 (53.49)	1
No	938	95.62	830 (88.49)	108 (11.51)	**7.69 (3.7 to 15.99)**	767 (81.77)	171 (18.23)	**4.4 (2.16 to 8.93)**
Any Negative Comments*								
Yes	13	1.33	6 (46.15)	7 (53.85)	1	7 (53.85)	6 (46.15)	1
No	968	98.67	843 (87.09)	125 (12.91)	**4.91 (1.25 to 19.23)**	780 (80.58)	188 (19.42)	**2.21 (0.59 to 8.3)**
Received explanation before procedures*								
Yes	829	84.51	748 (90.23)	81 (9.77)	**4.94 (3.16 to 7.7)**	709 (85.52)	120 (14.48)	**5.28 (3.54 to 7.87)**
No	152	15.49	101 (66.45)	51 (33.55)	1	78 (51.32)	74 (48.68)	1
Consented before procedures*								
Yes	787	80.22	714 (90.72)	73 (9.28)	**3.94 (2.58 to 6.02)**	670 (85.13)	117 (14.87)	**3.55 (2.44 to 5.16)**
No	194	19.78	135 (69.59)	59 (30.41)	1	117 (60.31)	77 (39.69)	1
Allowed to birth in preferred position (vaginal birth only)*	*N* = 777							
Yes	454	58.43	412 (90.75)	42 (9.25)	**2.14 (1.32 to 3.47)**	394 (86.78)	60 (13.22)	**2.07 (1.39 to 3.08)**
No	323	41.57	264 (81.73)	59 (18.27)	1	235 (72.76)	88 (27.24)	1
**Allowed a birth companion of choice**								
Yes	900	91.74	781 (86.78)	119 (13.22)	0.86 (0.39 to 1.88)	727 (80.78)	173 (19.22)	1.23 (0.65 to 2.33)
No	81	8.26	68 (83.95)	13 (16.05)	1	60 (74.07)	21 (25.93)	1
Separated from baby against wishes*								
Yes	261	26.61	210 (80.46)	51 (19.54)	1	177 (67.82)	84 (32.18)	1
No	720	73.39	639 (88.75)	81 (11.25)	**1.76 (1.15 to 2.71)**	610 (84.72)	110 (15.28)	**2.34 (1.63 to 3.37)**
Allowed to breastfeed on demand*								
Yes	780	79.51	689 (88.33)	91 (11.67)	**1.83 (1.15 to 2.91)**	649 (83.21)	131 (16.79)	**2.14 (1.44 to 3.16)**
No	201	20.49	160 (79.6)	41 (20.4)	1	138 (68.66)	63 (31.34)	1
Felt ignored by medical staff*								
Yes	118	12.03	40 (33.9)	78 (66.10)	1	22 (18.64)	96 (81.36)	1
No	863	87.97	809 (93.74)	54 (6.26)	**40.11 (22.87 to 70.34)**	765 (88.64)	98 (11.36)	**43.2 (24.21 to 77.22)**
Had to wait for long time for care*								
Yes	197	20.08	113 (57.36)	84 (42.64)	1	86 (43.65)	111 (56.35)	1
No	784	79.92	736 (93.88)	48 (6.12)	**11.41 (7.3 to 17.81)**	701 (89.41)	83 (10.59)	**11.28 (7.59 to 16.76)**
Had the opportunity to discuss any preferences or requests with the medical staff*								
Yes	671	68.4	638 (95.08)	33 (4.92)	**9.52 (5.99 to 15.12)**	619 (92.25)	52 (7.75)	**10.15 (6.93 to 14.86)**
No	310	31.6	211 (68.06)	99 (31.94)	1	168 (54.19)	142 (45.81)	1
Felt privacy was respected*								
Yes	804	81.96	745 (92.66)	59 (7.34)	**7.73 (5 to 11.95)**	694 (86.32)	110 (13.68)	**5.75 (3.91 to 8.48)**
No	177	18.04	104 (58.76)	73 (41.24)	1	93 (52.54)	84 (47.46)	1
Made joint decisions with medical staff*								
Yes	643	65.55	604 (93.93)	39 (6.07)	**6.11 (3.93 to 9.5)**	589 (91.6)	54 (8.4)	**7.66 (5.27 to 11.15)**
No	338	34.45	245 (72.49)	93 (27.51)	1	198 (58.58)	140 (41.42)	1

Separate models were fitted for each care practice. All models were adjusted for age, country of birth, marital status, education, income, parity, religion, level of religiosity, level of pregnancy risk, mode of delivery, and the occurrence of any intervention

Statistically significant AOR bolded

^*^Statistically significant at *P* ≤ 0.05

### Covid-19 related characteristics and practices

Table 4 presents Covid-19-related findings, including the associations between Covid-19-related care practices and perceptions of respectful and satisfactory care. Almost half of the participants were asked to wear a mask at some point during childbirth (44.44%). According to study participants, Covid-19-related hospital procedures were explained to more than half of participants (61.88%). This is the only Covid-19-related practice that was found to be significantly associated with the outcome variables. Having Covid-19 related procedures explained to participants increased by almost three times the likelihood of them reporting feeling that the care they received was respectful (AOR 2.89, 95% CI 1.91 to 4.36) and satisfactory (AOR 2.83, 95% CI 2.01 to 4).

**Table 4:** Covid-related measures and association with perceptions of care as respectful and satisfactory

	**Total**		**Respectful Care**			**Satisfactory Care**		
	N	%	Yes N(%)	No N(%)	AOR (95% CI)	Yes N(%)	No N(%)	AOR (95% CI)
**Asked to take a Covid test at the hospital**								
Yes	291	29.66	248 (85.22)	43 (14.78)	1	226 (77.66)	65 (22.34)	1
No	690	70.34	601 (87.1)	89 (12.9)	1.17 (0.76 to 1.8)	561 (81.3)	129 (18.7)	1.29 (0.9 to 1.85)
**Asked to wear a mask during childbirth**								
Yes	436	44.44	374 (85.78)	62 (14.22)	1	341 (78.21)	95 (21.79)	1
No	545	55.56	475 (87.16)	70 (12.84)	1.1 (0.74 to 1.64)	446 (81.83)	99 (18.17)	1.27 (0.91 to 1.78)
Covid-related procedure explained **								
Yes	607	61.88	556 (91.6)	51 (8.4)	**2.89 (1.91 to 4.36)**	527 (86.82)	80 (13.18)	**2.83 (2.01 to 4)**
No	374	38.12	293 (78.34)	81 (21.66)	1	260 (69.52)	114 (30.48)	1

Separate models were fitted for each care practice. All models were adjusted for age, country of birth, marital status, education, income, parity, religion, level of religiosity, level of pregnancy risk, mode of delivery, and the occurrence of any intervention

Statistically significant AOR bolded

^*^Statistically significant at *P* ≤ 0.05

## Discussion

This study sought to explore the associations between childbirth care practices and perceptions of care as satisfactory and respectful among women who delivered in Israeli Hospitals during the first six months of the Covid-19 pandemic. Building on the typology and community survey tool developed by WHO, this study assessed the perception of childbirth care among 981 women, accounting for 1% of all women who delivered in Israel within the specified time frame.

The findings of this study indicate that the majority of women who gave birth in Israeli hospitals during the first six months of the Covid-19 pandemic perceived the care they received as both respectful (86.54%) and satisfactory (80.22%). However, findings also revealed that 9.89% of women did not perceive the care they received as satisfactory or respectful, and a very high proportion (72.68%) of women reported experiencing at least one type of disrespectful care practice.

### Socio-Demographic Associations

The only association between socio-demographic characteristics and perceptions of care was found with parity. Primiparous women were significantly more likely to perceive their care as non-respectful or satisfactory, with 25% of these participants stating they were not satisfied. This finding is supported by other studies that found that women tend to perceive their childbirth experience negatively when their expectations are not met [42], and that such unmet expectations are more common at first birth compared to subsequent births [43]. This gap between expectations and reality can be a result of “unrealistic expectation for… an uneventful labor” prior to first childbirth [44].

### Obstetric associations

Our findings reveal that women who underwent an emergency C-section were significantly more likely to perceive their care as unsatisfactory than those who underwent an elective C-Sect. (28.7% compared to 14.61%). An Israeli study conducted in one tertiary hospital in 2021 evaluated the risk factors for a negative birth experience using the Birth Satisfaction Scale-Revised (BSS-R) questionnaire. The study, which focused on obstetric history and clinical factors, found that the proportion of emergency C-sections was higher in the negative birth experience group (56.9% vs. 43.1%, P < 0.001), suggesting that undergoing an emergency C-section was associated with an increased likelihood of a negative experience [44]. In addition, a systematic review of subjective perceptions of childbirth experience found that emergency C-sections and instrumental births were associated with more negative experiences [42]. Our study also found similar findings regarding instrumental births, with 30.12% of women whose birth involved forceps or vacuum reporting non-satisfactory care, compared to 18.82% of those whose vaginal birth was not instrumental. This finding is also supported by an Israeli study on skin-to-skin contact and birth satisfaction by mode of birth that found that women who had an instrumental birth reported lower birth satisfaction compared to those who had a vaginal non-instrumental birth [45]. A common factor might be that births requiring instrumental or surgical intervention are, almost by definition, more complex, and likely to be more stressful for both the woman and her medical care providers.

### Childbirth care practices associations

The most prevalent types of disrespectful practices found in this study can be categorized into two of the themes identified by Bohren and colleagues (2015): failure to meet professional standards of care (lack of informed consent, breach of privacy, and feeling ignored), and poor rapport between women and medical staff (denial of preferred birth position, forced separation from newborn, lack of opportunity to discuss preferences or concerns, and absence of joint decision-making). The magnitude of the effects of the various mistreatment practices on women’s perception of respectful and satisfactory care reveals that women place high value on feeling seen and heard by the medical staff charged with their care. Women who did not feel ignored, and those who felt that they had the opportunity to discuss their preferences with their medical staff, were significantly more likely to perceive their care as respectful and satisfactory. A systematic review that explored risk and protective factors for women’s childbirth experience and birth satisfaction found that feeling unseen or unheard during birth was associated with a more adverse childbirth experience [42].

Our findings suggest that greater involvement and perceived control over the birth process are associated with an increased likelihood of perceiving care as respectful and satisfactory by birthing women. These findings are supported by a multitude of other studies, including a longitudinal Israeli study that used physical, emotional, and cognitive factors to assess birth satisfaction based on frameworks of stress and control. Similarly to our study, it found that greater control over the birth environment, including medical staff actions and interventions, predicted positive emotions and better-perceived care [46]. Similar results were detailed in a systematic review that found that “the higher the perceived control, the better the subjective experience of childbirth” [42]. The comparative importance of childbirth care practices related to communication versus other care practices may reflect women’s perception that behavior of medical staff is changeable, while some hospital policies may not be. This appears to hold true with communication about Covid-19 policies, which had a greater influence on reports of respectful care and satisfaction than some of the policies themselves.

The study findings also reveal a substantial discrepancy between women's reported disrespectful experiences during childbirth and their overall perceptions of the care they received as both respectful and satisfactory. While a considerable majority expressed positive perceptions, most participants (72.68%) reported they experienced at least one disrespectful care practice. This inconsistency merits exploration through various potential explanations.

One possible explanation might be that while women recognize and acknowledge the occurrence of disrespectful practices, they do not perceive them to be a form of disrespectful care. Disrespectful practices may have been internalized as acceptable or normal to the extent that their occurrence has little to no effect on women’s perception of the care they received as respectful or satisfactory [47–49]. This may reflect an assimilation of such practices within the healthcare setting, rendering certain care practices as commonplace and expected. This perspective aligns with existing research suggesting that satisfaction metrics could inadvertently overlook normalized mistreatment occurrences, thereby masking concerns that are perceived as routine [50].

The overarching influence of socio-cultural norms is also a part of the normalization of disrespectful care practices. A study of obstetric violence in the Eastern Mediterranean posited that mistreatment often stems from deeply rooted patriarchal norms, resulting in behaviors that may go unnoticed by healthcare providers. Moreover, these societal norms contribute to a broader framework in which abusive behaviors become embedded [51].

In a philosophical analysis of obstetric violence, the authors showed that women are shamed for desiring a humane birth where they are respected and cared for instead of focusing exclusively on the health of their newborn. The shame described here may provide another explanation as to why study participants who reported disrespectful care still reported that they perceived the care they received as respectful and satisfactory [52].

An additional explanation relates to the adaptive nature of women's expectations. A study of the influence of expectations on the perception of the childbirth experience conducted in Australia discussed how women may recalibrate their childbirth expectations to better align with the realities of a healthcare system prioritizing positive health outcomes over the birthing experience itself. This adaptive response may arise from cognitive dissonance, prompting individuals to align their perceptions with healthcare norms to minimize emotional discomfort [43]. Furthermore, perceptions may change in the time between experience and data collection [50]. In this study, women reported their satisfaction five to ten months after childbirth, having had time to process and share their experiences and possibly change their perceptions of care. Positive health outcomes of childbirth for both women and newborns may also contribute to the gap between disrespectful care practices and perceptions of care, particularly as time passes [53].

The context of the Covid-19 pandemic may also contribute to the gap between the occurrence of care practices and perceptions of care. Media coverage and the public discourse at the time, potentially portraying more negative childbirth experiences worldwide, could have led to tempered expectations, resulting in perceptions of care that surpassed initial expectations [54]. Israeli studies of pregnant women’s anxiety during the first and second waves of the Covid-19 pandemic revealed that childbirth anxiety was higher in the first wave (March–April 2020), compared to the second wave (September–October 2020) [55]. It is plausible that expectations were lowered to a degree, that even with some disrespectful care practices, the overall experience of women was more positive than they had expected. A number of studies have been published assessing childbirth experiences in high-resource countries during the first few months of the Covid-19 pandemic. An Australian study revealed that most birthing women surveyed were satisfied with the maternity care they received despite changes to its delivery due to the pandemic. The authors posited that women recognized that their maternity providers were doing their best despite a “rapidly evolving situation” [40]. A study conducted in the United States found that overall, women who gave birth in the first few months of the pandemic mostly agreed with the description of the birth experience as satisfying [56]. Studies in New York and Italy, two of the first epicenters of the pandemic [57], compared satisfaction of births between March and May 2020, to pre-pandemic levels of satisfaction. The studies revealed that while in New York, satisfaction dropped from 58.6% to 43.1% [58], in Italy, satisfaction levels remained similar [59]. A large study of birth experiences during the first year of the pandemic in 12 European countries provides valuable data for comparison with the findings of this study [60]. The European study reported separate outcomes for women who underwent labor and those with a pre-labor C-section, finding that 42.8% of participants with spontaneous vaginal birth could not choose their birth position, compared to 41.57% in this study; that 7% and 14.3% of women were not allowed to stay with their baby as they wished, compared to 21.63% of women in this study; and that 18.2% and 20.5% reported lack of privacy, compared to 18.04% in this study. From the providers’ perspective, a global survey of health workers found that those from high-resource countries reported higher levels of perceived somewhat or substantially lower ability to provide respectful maternity care during the pandemic compared to health workers from middle or low-resource countries [12].

### Implications for research and practice

The findings of this study provide new insights as to what contributes to women’s perception of respectful and satisfactory maternity care, even during an unprecedented public health crisis. The factors found to be associated with a positive perception of maternity care illustrate the importance of communication and trust between birthing women and their medical staff, as well as respect for women’s autonomy and control. Also evident from these findings is that even when disrespectful practices occur during childbirth, women may still perceive the care they received as satisfactory and respectful. This does not mean, however, that disrespectful practices should not be addressed on both the interpersonal and health facility and policy levels. Women should be aware of their rights in health care facilities, and have high expectations for respectful care [61].

Quality maternity care should be woman-centered and ensure that the birthing woman is supported in her preferences during the childbirth experience. In Israel, where almost all births take place in hospitals, the factors that result in a positive perception of care should be integrated into routine maternity care. In addition, it is crucial, particularly during rapidly evolving crises such as the Covid-19 pandemic, that WHO and Ministry of Health guidelines on maintaining respectful care be implemented across the health system.

Future research on respectful maternity care in Israel should strive to expand the understanding of how different sub-groups within the population perceive respectful and satisfactory care, and what weight they place on the various factors that contribute to such care. In addition, future research should explore potential interventions to address the disrespectful practices, including those found in this study.

### Strengths and limitations

The key strengths of this study include its novelty in exploring childbirth care practices and their associations with perceptions of care in Israel. An additional strength is the use of the WHO community survey tool aimed at measuring how women are treated during childbirth. However, the adaptations made to the tool in order to allow for self-administration and utilization in the Israeli context can be regarded as a limitation. The study included every hospital in Israel with a maternity ward, and reached a relatively high sample size, which accounts for close to 1% of all births that occurred in Israel during the specified period. Another strength of this study was its measurement of not only mistreatment care practices but a broader scope of both disrespectful and respectful care practices and their associations with perceptions of care. Given the multifaceted nature of respectful maternity care, multiple measures are necessary for driving meaningful quality improvements.

Our study has some limitations. First, It is essential to acknowledge the potential implications stemming from the underrepresentation of women who belong to smaller social groups in Israel, particularly Arab, Palestinian, and Bedouin women (of all levels of religiosity), immigrant women, and Ultra-orthodox Jewish women. These underrepresentations may be a result of the convenience sampling method used, and the use of the Hebrew language as the sole language of the survey, which excluded women who were not literate in Hebrew, mainly Arab and Bedouin women, as well as migrant women. However, according to a 2019 study, 45.8% of Arab women in Israel have good or above command of Hebrew [62]. As this percentage does not account for age, it is likely that the percentage among women of reproductive age is higher. Members of these groups, as well as Ultra-Orthodox women of all religions, are also less likely to be social media users, where study recruitment occurred.

The experiences and perspectives of women belonging to the above underrepresented groups may differ from those captured in our study, and thus, our conclusions should be interpreted within this context. While this study did not find significant variations based on religion, several studies conducted in Israel reveal that women who belong to minority groups can have unique childbirth care experiences and perceptions. Identity, location, type of hospital, and the medical staff can all play an important role in perceptions of care among minority women. A study that explored racial maternal separation in Israeli maternity wards found that it mainly targeted religious Muslim Palestinian-Arab women, some of whom perceived it as discrimination, while others normalized it as a preference [63]. Several other studies reveal the importance of exploring minority women’s experiences. Studies have found that Arab women have a higher rate of traumatic birth compared to Jewish women [64]; that while pain intensity self-assessments during the initial active labor phase were similar between Jewish and Bedouin Arab women, Jewish medical staff tended to underestimate pain felt by Bedouin women [65]; and that during the Covid-19 pandemic, pregnant Arab women had a higher rate of childbirth anxiety, and more Covid-19 related concerns for the fetus [66].

Other limitations and biases that may have skewed the study’s results should be noted. The design of the study as a retrospective cross-sectional study without baseline data results in an inability to compare pre-pandemic data to the study findings. Instead, this study provides a description of the perceptions of maternity care in Israel during the Covid-19 pandemic that allows for comparison with similar studies conducted in other settings and over time in Israel. The study design may have also led to recall bias as data was collected between four and ten months following childbirth, although significant events such as childbirth, often have better recall than more frequent or mundane events.

## Conclusions

The majority of women who participated in this study perceived the maternity care they received as both respectful and satisfactory. The study identified several key factors that are associated with how women perceive their care, including respectful and disrespectful care practices, with many important factors related to communication and trust. Understanding what women associate with a positive childbirth experience is key to improving respectful maternity care in Israeli hospitals, which is needed to ensure the physical and mental health and well-being of both women and newborns. The findings of this study can be used to inform future work and research at both the national and facility levels.

## Data Availability

The datasets used and analyzed during the current study are available from the corresponding author on reasonable request.

## Abbreviations

AOR: Adjusted odds ratio
C-section: Cesarean section
CI: Confidence interval
WHO: World Health Organization
